# Analysis of the impact of healthcare support initiatives for physically disabled people on their access to care in the city of Saint-Louis, Senegal

**DOI:** 10.1186/s12913-017-2644-y

**Published:** 2017-12-04

**Authors:** Diarra Bousso Senghor, Oumar Diop, Issa Sombié

**Affiliations:** 1 Department of Geography, College of Arts and Human Sciences, Gaston Berger University, Saint-Louis, Senegal; 2 Higher Institute of Population Sciences, Ouaga 1 University, Ouagadougou, Burkina Faso

**Keywords:** Disability, Health care, Access to care, Senegal, Urban

## Abstract

**Background:**

People with disabilities represent approximately 6% of the Senegalese population. They face significant barriers to accessing health care. Although several initiatives have been implemented to improve access to health care for this vulnerable population, few studies have examined the effects of these initiatives. We conducted a mixed methods study in three neighborhoods in Saint-Louis City (Senegal) to assess the impact of health systems and social assistance programs aimed at improving access to health care for people with disabilities.

**Methods:**

Data were collected from 105 people living with disabilities aged 1–49 years (or their caregivers). Interviews were also conducted with key stakeholders in the health and welfare sectors. Global Positioning System (GPS) coordinates of all the health and social services within the city were obtained. We also conducted observations in the main regional hospital, the district health center and three level-one health facilities to assess physical accessibility as well as interactions between patients living with disabilities and health and social workers. Descriptive and multivariate analyses were performed using Sphinx software. Spatial data were used to make cartographic representations of the proximity to basic social services using Arc GIS software.

**Results:**

Seventy-nine percent of survey respondents reported difficulty obtaining treatment. Key barriers to care included the high cost of care, as well as ill-treatment by health workers. Limited human resources and low levels of financial support, combined with logistical challenges were reported to hamper the success of social welfare initiatives that aim to facilitate access to health care for people with disabilities.

**Conclusion:**

Our results suggest that initiatives to increase access to health care among people with disability in Saint-Louis have had limited impact. Study findings underscore the importance of strengthening social assistance schemes within the health system and the need for social workers and health workers to collaborate to improve access to health care for people with disabilities.

## Background

People with disabilities (PWD) represent approximately 6% of the Senegalese population. They face significant barriers to accessing health care services. According to Handicap International [1], majority of PWD in Senegal live in poverty and are unable to meet their health care needs. The inadequate number and unequal distribution of health care facilities and the near absence of social welfare programs further limit PWD’s access to health care [1]. Several health systems and social assistance initiatives have been implemented in the country to improve access to health care for PWD. For example, the national policy on universal health coverage implemented in September 2013 includes an extension of social protection programs (health insurance schemes and free care) to vulnerable groups including PWD. Further, the Social Guidance Law implemented in October 2012 extends access to health care for PWD without medical insurance by covering health care fees, orthopedic equipment, hearing aids and necessary technical aids. However, several years after the launching of these initiatives, PWD in Saint-Louis City continue to face significant barriers to accessing health care.

Gueye and Seck [2] suggest that the human resources for health crisis, as well as limited material and financial resources hamper the availability of quality health care, particularly for PWD. Further, dissatisfaction with the quality of care [3], poor treatment by health care providers [3, 4], unequal distribution of health care structures and health personnel [5, 6], as well as the lack of training for health care providers [7–9] limit both physical and social access to care for all, but more so for PWD.

Improving health care access for PWD is complex because it requires inputs from multiple sectors [10]. For example, studies conducted in France reveal that the lack of information on medico-social devices, limited time for consultation, and poor coordination between actors in the health sector and those in the social assistance sector hamper the provision of adequate health care for patients living with disability in urban areas [11]. Further, effective support of PWD is limited by perceptions that disabilities are a consequence of personal deficiencies and not a product of failure of the Senegalese society and environment [12]. As a result of these perceptions, decision-makers may fail to provide the equipment and resources needed for health, orthopedic and social services to adequately care for people with disabilities. Limited awareness of existing initiatives aimed at supporting PWD also limits the use of services [1].

Although existing studies provide useful information about the factors that may limit PWD’s access to health care, they do not examine the effects of recent initiatives aimed at improving access to care for PWD. In this study, we used mixed methods to evaluate the impact of initiatives aimed at increasing access to health care for PWD in Saint-Louis, Senegal. We also assessed factors that may hinder the success of these initiatives.

## Methods

### Study setting

**Fig. 1:**
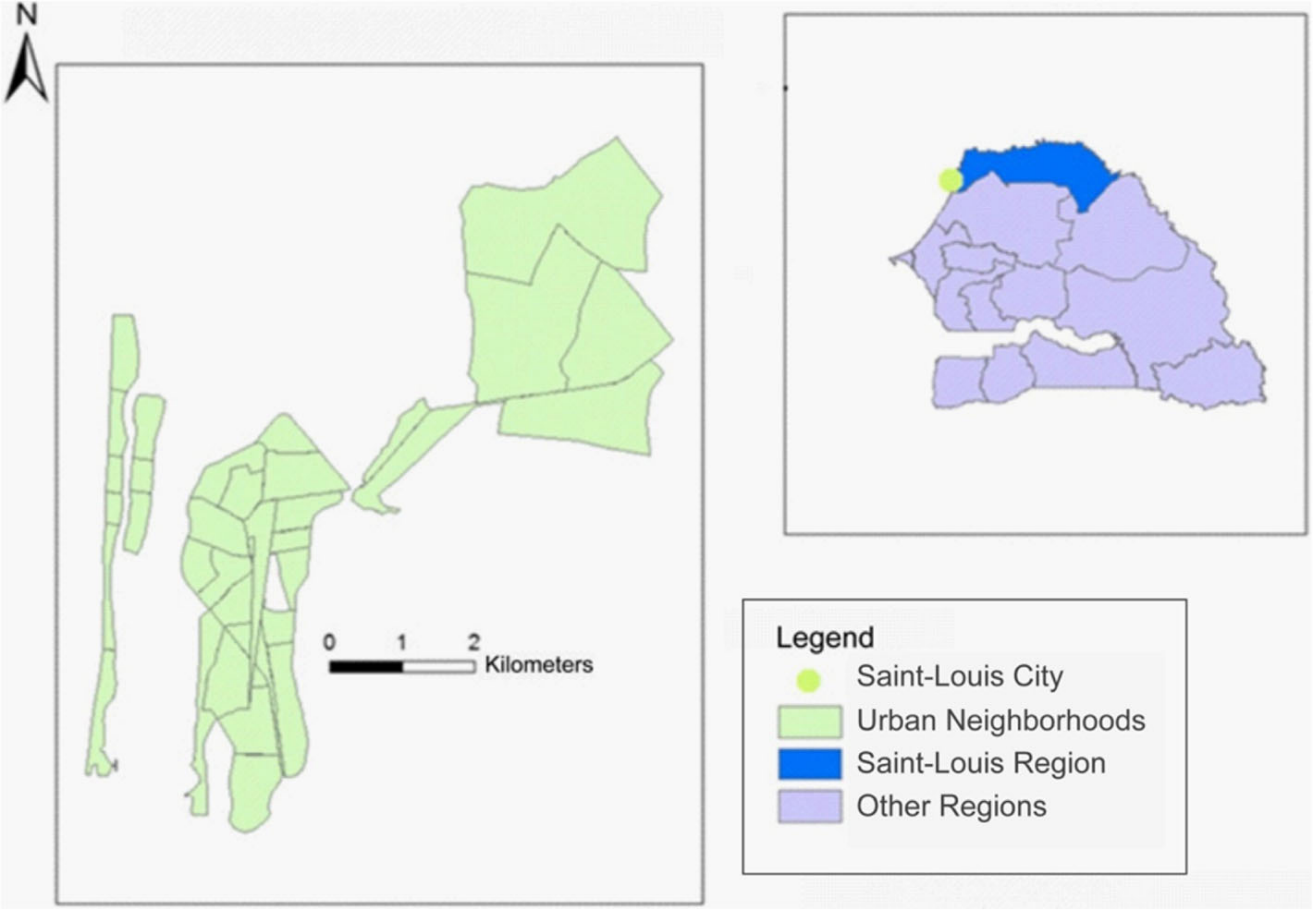
Geographical map of Senegal showing Saint-Louis City, Senegal

The study was conducted between September 2015 and September 2016 in Saint-Louis City, the capital of the Saint-Louis Region (Fig. 1). The city covers an area of 4579 ha and is subdivided into 33 neighborhoods with 203,881 inhabitants [13]. The city is part of the health district of Saint-Louis. Most of the district’s health infrastructure is concentrated in the city, which hosts 13 out of the 18 health stations in the district as well as the Saint-Louis Regional Hospital. The city also houses the department of social action as well as three centers for promotion and social reintegration that provide various social services.

We purposively selected three neighborhoods in Saint-Louis City: the Langue de Barbarie, the suburb of Sor, and the peri-urban neighborhood of Bango. The downtown area, which mainly comprises commercial or administrative offices, was excluded. The selected neighborhoods reflect varying characteristics in terms of location in the city, the types of roads (paved, unpaved, winding, etc.), the proximity to health services, and structures for social care, including access for PWD.

### Sampling

In each neighborhood, we identified all people living with physical disabilities (motor, visual and albinism) who were aged 50 years or younger. We excluded people with mental or sensory disabilities that would limit their ability to participate in the survey. Because of the significant association between old age and disability [13], we excluded respondents older than 50 years. We also conducted key informant interviews with five health workers (an orthopedist, a physiotherapist, an ophthalmologist, a gynecologist and a general practitioner), four social workers (two in the hospital and two in the department of social work), and two mutual health insurance staff.

### Data collection procedures and tools

Survey data were collected via face-to-face interviews using a questionnaire with open- and close-ended questions. For children with disabilities, we interviewed one of their caregivers (mostly mothers and a few fathers and grandmothers). The questionnaire elicited information on participants’ socio-demographic characteristics; socio-economic status; mobility; care needs; use of health care (frequency, types, place); cost of care; possession of an equal opportunities card (EOC)—an identity card that provides PWD certain privileges and support in accessing healthcare, employment, training and other public services; access to social assistance; access to information; as well as perceptions of the quality of health care including reception, relationships with health personnel, and satisfaction of care needs. Interviews were conducted in the respondents’ homes.

Qualitative data were collected using a semi-structured interview guide that elicited information on barriers to health care among PWD and perceptions of the implementation of initiatives that aim to improve access to health care for PWD. We also conducted observations in the main regional hospital, the district health center and three level-one health facilities to assess physical accessibility as well as interactions between patients living with disabilities and health and social workers. Finally, we collected Global Positioning System (GPS) coordinates of all the health and social services in the city.

### Ethical considerations

Ethical approval for this study was granted by the Commission of the Doctoral School of Human Sciences and Society at the Gaston Berger University, Saint-Louis. All participants were briefed about the objectives of the study, the benefits and risks of participation, and the precautions taken to ensure confidentiality. All participants consented to participation in the study.

### Analysis

The survey data were analyzed using Sphinx software. We computed descriptive statistics aggregated by location. Spatial data were analyzed using Arc GIS software to generate maps illustrating the geographical distribution of health care and social assistance services. Qualitative data were transcribed and coded into themes capturing the key domains of interest.

## Results

### Participants

The survey sample comprised 105 persons with disabilities, 49 (47%) of whom were female. Their ages ranged from 1 to 49 years. The sample included 34 children who were aged 16 years or younger and dependent on a parent or guardian.

### Access to health care

#### Domains of health care access for people with disabilities

Survey respondents underscored the significant challenges faced by PWD in Saint-Louis in accessing health care. These challenges were not only related to physical and financial accessibility, but also to social or relational accessibility. Table 1 summarizes survey respondents’ ratings of various domains of health care accessibility.

**Table 1:** Participants’ ratings of key domains of health care access (*N* = 105)

Domains of health care access	Acceptability				
	Not at all	Somewhat	Moderate	Very	Total
Proximity to a health care facility	28%	19%	30%	23%	100%
Cost of health care	53%	9%	27%	11%	100%
Reception at health facility	48%	11%	20%	21%	100%
Quality of interactions with health care providers	35%	28%	23%	14%	100%

**Fig. 2:**
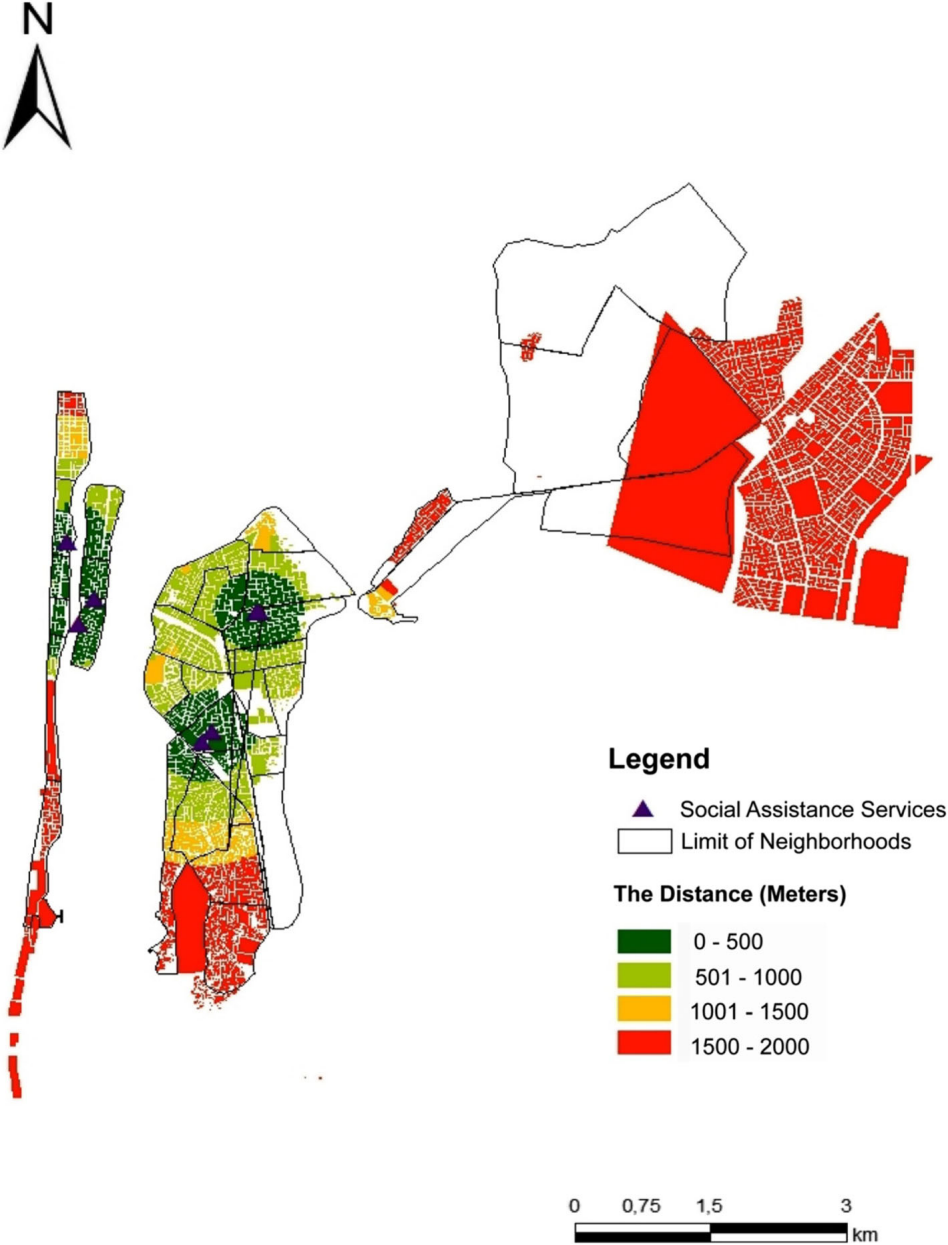
Distances to social assistance services in Saint-Louis City, Senegal

Twenty-eight percent of survey respondents reported that the distance to health care facilities (physical access) was not acceptable at all. Analysis of the spatial data showed that majority of inhabitants lived less than 1 km from a health facility or social assistance provider, on average. However, there were significant variations based on location with distances to service providers exceeding three kilometers in the southern and eastern parts of the city. Welfare or social assistance services were located much further than health facilities (Fig. 2) with distances exceeding 1 km in most parts of the city and more than four kilometers in some parts of the city.

Based on the responses to open-ended questions in the survey, physical accessibility was hindered by the lack of adaptive infrastructure (e.g., elevators) and devices to facilitate mobility; the lack of specialized care in many facilities, which forced patients to seek care in better-equipped facilities that were further away; and the lack of adequate means of transport, especially public transport. For example, one 46-year-old man living in Bango noted that he sometimes had to wait for long durations (even up to 2 hours) before finding a taxi operator who was willing to carry him. A 39-year-old woman living in the same neighborhood recounted her difficulties visiting a gynecologist noting:

*I could not reach the office of the gynecologist because it is located upstairs. I was dependent on the agents at the reception who were to carry me and help me go up and down; that is very humiliating for a woman. Not to mention that they expect me to motivate them by giving them some money.*

Physical accessibility was also a challenge for caregivers. One parent reported that he experienced health problems because of having to carry his child around. A young woman with severe motor disabilities also stated, *“It is difficult for my mother to take me to the health department with my wheelchair because she is old and the ways are not adapted.”*

As shown in Table 1, 53% of survey respondents reported that the cost of health care was not acceptable at all, while only 11% reported that the cost of health care was acceptable. Survey respondents noted that PWD face significant challenges accessing health care due to their limited financial resources and the lack of strong social assistance programs to enable them meet health care costs. Few of the PWD surveyed had a source of income and among those who did, wages were typically low. Further, only 19% of all survey respondents had medical insurance cover as the majority of those employed were working in the informal sector. Survey respondents recounted experiences of being denied care because they lacked money to pay for services. For example, a woman whose 9-year-old son had cerebral palsy described how she was denied care because she did not have money to pay. She explained that she had to wait for more than an hour at the entrance with the child on her back and eventually returned home without receiving any care. Others noted that they were forced to beg for money or take loans to pay for health services. Many survey respondents also reported that they were unable to purchase prescribed medication due to the high cost of medicines.

On the social and relational level, nearly half of the survey respondents (48%) reported that the reception they received in the health facility was not acceptable at all, while about a third (35%) rated the quality of interactions with healthcare professionals similarly. Many respondents reported unwelcoming reception while others stated that they were not adequately informed and oriented about where services were offered and hence spent time looking around for the right service location. Survey respondents also highlighted long waiting times and reported that they were occasionally denied priority, despite having an EOC, which grants them priority access. Some respondents also felt that hospital workers were insensitive to the needs of PWD and were only interested in money.

### Health care utilization

Sixty-six percent of PWD had sought care for their most recent illness. Table 2 summarizes the proportion of PWD seeking care in a health facility for their most recent illness by ratings of key domains of health care access. Only 55% of PWD who rated their proximity to health facilities as *not acceptable at all* sought care in a health facility for their most recent illness compared to 75%, 72% and 68% of those who rated their proximity to a health care facility as *somewhat*, *moderately*, or *very acceptable*, respectively. Similar differences were observed for the other domains of access with a smaller proportion of PWD who viewed each aspect of access unfavorably reporting that they sought care in a health facility compared to those rating the domain favorably. Overall, 53% PWD reported that they had to seek alternative care options because of the challenges faced in accessing health care services (not shown).

**Table 2:** Proportion of survey respondents seeking care in a health facility for most recent illness by ratings of key domains of health care access (*N* = 105)

Domains of health care access	Acceptability rating	Care sought for most recent illness	
		Yes *n* = 69	No *n* = 36
Proximity to a health care facility	Not at all	55%	45%
	Somewhat	75%	25%
	Moderate	72%	28%
	Very	68%	32%
Cost of health care	Not at all	63%	37%
	Somewhat	66%	34%
	Moderate	72%	28%
	Very	75%	25%
Reception at health facility	Not at all	54%	46%
	Somewhat	46%	54%
	Moderate	88%	12%
	Very	74%	
Quality of interactions with health care providers	Not at all	62%	26%
	Somewhat	85%	15%
	Moderate	87%	13%
	Very	100%	0%

The narratives from PWD and their caregivers suggested that the challenges they faced in accessing health care resulted in some of them choosing not to seek care. Others reported that they instead sought medication from pharmacies—a cheaper and often more accommodative alternative. The use of pharmacies was highlighted by one participant who stated, “*I preferred to go to the pharmacy, which is near my house, there I was given credit for some of the medicines, because I did not have enough money and I was afraid that at the health center the costs would be expensive*.” Similarly, a mother of two children with cerebral palsy stated, “*They both had the flu and I did not have enough money to take them to the hospital so I went to the pharmacy to buy a syrup for them*.”

With respect to social or relational accessibility, some respondents stated that previous negative experiences made them unwilling to seek care in health facilities, one PWD, for example, stated, “*One day, I was very ill but I did not have any money. I went to the health center, I asked to be exempted from my care at the social worker level. The exemption was granted to me but it was followed by vexing reproaches. So I vowed to give up health care*.”

### People living with disabilities’ perceptions about initiatives to improve their access to health care

Majority of PWD or their caregivers felt that many of the initiatives aimed at improving PWD’s access to health care were unsatisfactory and argued that if the social assistance system was well-functioning they would not have challenges accessing health care. Narratives from survey respondents revealed multiple reasons for poor implementation and low effectiveness of the initiatives. One respondent alluded to limited funding and bureaucratic processes stating, “*When we apply to the social service, the procedure is slow and often without results. According to them, these structures have no resources and are there only for the sake of being there*.” These sentiments were echoed by another respondent who stated, “*At the hospital the social worker imposes on us tiring procedures that do not lead to anything*.”

Limited awareness of and publicity around existing services were also highlighted as reasons for the low effectiveness of the programs. We observed that 51% of survey respondents either did not know that these initiatives existed, did not understand their purpose, or had not used the services. According to one respondent who was a member of an association of PWD, there were no initiatives to inform PWD about the rights and benefits granted to those possessing an EOC.

Access to services was also perceived to be based on personal relationships with social workers. Other survey respondents also felt that the shortcomings of these initiatives were mainly because the people chosen to represent PWD were only concerned with their own interests and did not push for decision-makers to respect commitments made to PWD. As a 32-year-old man living in Langue de Barbarie noted, “*Everyone is looking after [their own] interests that is why decision-makers do not consider us seriously*.”

Survey respondents also noted that the delivery of services did not take into account the needs of PWD. One respondent for example stated, “*We were not involved to find out what we really needed. They have taken these initiatives for the sake of taking it but not to really solve our problems*.” Others noted that social service offices in hospitals were located upstairs making it difficult for PWD to access these services. As one survey respondent suggested, “*It is a strategy to discourage us because they do not want to receive many requests*.”

#### Key informants’ perceptions about initiatives to improve access to health care for people living with disabilities

Key informants acknowledged that the initiatives aimed at improving PWD’s access to health care had multiple shortcomings. First, health workers reported that the government had not put in place adequate systems to enable health facilities meet PWD’s needs. The health workers noted for example that the absence of a reimbursement agreement with the government meant that health facilities were unwilling to provide free care to patients with an EOC. As a result, they argued that many of the difficulties experienced by patients with disabilities at the health facility level were related to PWD’s inability to pay for services. Relatedly, key informants also indicated that while the government was committed to facilitating the registration of PWD in health insurance programs, the financial support was insufficient and often delayed, and the contributions from members inadequate to support the operation of these insurance programs. Key informants also noted that the administrative process of receiving social assistance was lengthy and complex.

With respect to PWD’s perceptions of health workers’ negative attitudes, health care workers noted that some patients were unaware of the operating realities of the hospital and expected more than health workers were able to offer them, while other patients were prejudiced against health workers. They also noted that their heavy workloads affected their interactions with patients and acknowledged that a few staff lacked awareness or experience handling patients with disabilities. Observations in some of the health facilities showed that health workers were often overwhelmed by the large number of patients waiting for them. As a result, consultation times were often short, which was frustrating for some patients. Further, we observed that PWD were rarely given priority when there were many patients.

Health workers also highlighted the lack of support from facility administrators as a challenge. Specifically, they commented on the inadequacy of equipment adapted to PWD and linked this to the unwillingness of facility administrators to invest in infrastructural changes given the relatively small number of patients with disabilities. For example, one worker noted that in 4 months only three women with motor disabilities had delivered a child in the hospital. Another health worker who was stationed in an orthopedic unit explained that hospital administrators viewed this unit as a “*costly and unprofitable service*”, which meant that few resources were allocated to it and that the unit suffered from a shortage of skilled staff.

Social workers also noted that the lack of resources impeded the delivery of social assistance programs. For example, they stated that the social assistance system had a very tight budget and the lack of logistical and human resources made it difficult for them to adequately provide services. One social worker in a hospital further explained that their low budgetary capacity did not allow them to grant exemptions, which were in his opinion a “luxury”. Social workers also noted that their awareness of the limitations of the services also led them not to inform PWD about services offered.

Finally, regarding delays in the allocation of the equal opportunity cards, social workers highlighted the limited geographical spread of social assistance offices, which made it difficult for PWD to obtain the cards. They further noted that the filing process was delayed by agents’ limited ability to communicate with people living with sensory disabilities. They explained that people with such disabilities were often asked to return at another time if a translator was not available or advised to return with a close relative who could answer the agent’s questions. Key informants also noted that the process of receiving social assistance was lengthy and complex because of the multiple approvals needed and the need to verify applicants’ eligibility.

## Discussion

In this study, we assessed the impact of programs aimed at increasing access to health care for PWD in Saint-Louis, Senegal and investigated the factors that may hinder the success of these initiatives. We found that PWD in Saint-Louis City have limited access to care despite the programs implemented to facilitate access. The most important factors impeding access were the cost of care, physical inaccessibility, and poor relationships with health workers. The significance of financial barriers to health care access mirrors findings in other studies. For example, Gobbers [14] has demonstrated that the availability of financial resources is a key factor in the utilization of health services. Similarly, Gulliford [15] notes that financial and physical accessibility, as well as acceptability of care influence the use of health care services, even in cities with abundant supply. Pichetti [16] further suggests that limited health utilization among PWD is attributable to their disadvantaged social situation. According to Handicap International [1], majority of Senegalese PWD live in poverty and are unable to meet their health care needs. These results suggest that need to strengthen and expand social assistance programs so that PWD, especially those with limited resources, can access health care.

Similar to other studies [3, 4], we found that poor relationships between health care, social workers and patients with disability resulted in some PWD opting not to seek care. In their study in five capitals of West Africa, Jaffré and Olivier de Sardan [3] also found that patients with disability were often poorly treated by health workers. Similarly, Gruénais [4] reported a lack of consideration of patients with disabilities among health personnel in public health facilities in sub-Saharan Africa. These findings underscore the importance of strengthening the capacities of health personnel and social workers to respond to the needs of PWD and providing an enabling environment for these professionals to deliver quality care.

Although several initiatives have been developed to support PWD’s access to health care in Senegal, our results suggest that the implementation of these initiatives is limited by a range of factors that cut across multiple sectors. These include a weak social welfare system coupled with limited budgets, human resource constraints, and the lack of adequate facilities. Josse [10] reports that a multi-sectoral approach is necessary to enable PWD to benefit from preventive and curative care, rehabilitation and other social programs. Thus, efforts to improve PWD’s access to healthcare, should target key sectors including social welfare, finance, and health.

Study findings should be interpreted in light of the following limitation; the survey was limited to PWD living in selected neighborhoods in the city and cannot be considered to be representative of the entire city of Saint-Louis. Nevertheless, the data shed important light on the challenges faced by PWD in accessing health care services in an urban context that may be relevant for similar cities in sub-Saharan Africa. Further studies that examine factors associated with access to health care among PWD in other settings are warranted.

## Conclusions

Although the government of Senegal has implemented several initiatives to improve access to health care for PWD, our results suggest that these initiatives have had little success. Key impediments to their success are the weak social welfare system coupled with the lack of adequate facilities and resources in health facilities. Consequently, PWD are unable to afford health care costs and often have poor relationships with health and social workers. Our results underscore the importance of strengthening, streamlining, and adequately funding social assistance schemes that will improve access to health care for PWD. Our results also highlight the need for social workers and health workers to work collaboratively and for better training of providers to offer patient-centered care.

## Abbreviations

EOC: Equal opportunity card
GPS: Global Positioning System
PWD: People living with disabilities
